# Functional near‐infrared spectroscopy in toddlers: Neural differentiation of communicative cues and relation to future language abilities

**DOI:** 10.1111/desc.12948

**Published:** 2020-03-20

**Authors:** Elizabeth G. Smith, Emma Condy, Afrouz Anderson, Audrey Thurm, Stacy S. Manwaring, Lauren Swineford, Amir Gandjbakhche, Elizabeth Redcay

**Affiliations:** ^1^ University of Maryland College Park MD USA; ^2^ National Institute of Child Health and Human Development Bethesda MD USA; ^3^ National Institute of Mental Health Bethesda MD USA; ^4^ University of Utah Salt Lake City UT USA; ^5^ Washington State University Spokane WA USA

**Keywords:** brain development, fNIRS, gesture, language, speech, toddler

## Abstract

The toddler and preschool years are a time of significant development in both expressive and receptive communication abilities. However, little is known about the neurobiological underpinnings of language development during this period, likely due to difficulties acquiring functional neuroimaging data. Functional near‐infrared spectroscopy (fNIRS) is a motion‐tolerant neuroimaging technique that assesses cortical brain activity and can be used in very young children. Here, we use fNIRS during perception of communicative and noncommunicative speech and gestures in typically developing 2‐ and 3‐year‐olds (Study 1, *n* = 15, *n* = 12 respectively) and in a sample of 2‐year‐olds with both fNIRS data collected at age 2 and language outcome data at age 3 (Study 2, *n* = 18). In Study 1, 2‐ and 3‐year‐olds differentiated between communicative and noncommunicative stimuli as well as between speech and gestures in the left lateral frontal region. However, 2‐year‐olds showed different patterns of activation from 3‐year‐olds in right medial frontal regions. In Study 2, which included two toddlers identified with early language delays along with 16 typically developing toddlers, neural differentiation of communicative stimuli in the right medial frontal region at age 2 predicted receptive language at age 3. Specifically, after accounting for variance related to verbal ability at age 2, increased neural activation for communicative gestures (vs. both communicative speech and noncommunicative gestures) at age 2 predicted higher receptive language scores at age 3. These results are discussed in the context of the underlying mechanisms of toddler language development and use of fNIRS in prediction of language outcomes.

## Graphical abstract

Changes in blood oxygenation in the frontal lobe varied in toddlers depending on whether they saw gestures or heard speech, and depending on whether those gestures or speech were meaningful or nonmeaningful. This varied between 2‐ and 3‐year‐olds, and for 2‐year‐olds, predicted later language measures.

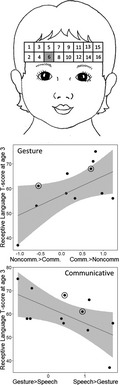

**RESEARCH HIGHLIGHTS**

- We use fNIRS in 2‐ and 3‐year‐olds to show left‐lateralized, differential processing of speech and gestures
- Compared to 3‐year‐olds, 2‐year‐olds showed differential processing of gestures and speech in right medial frontal cortex
- Functional activation in right medial areas in 2‐year‐olds predicted receptive language scores at age 3

## 1 INTRODUCTION

While the co‐emergence of speech and gestures in the first several years of life is well documented (Bates & Dick, 2002), research on the developmental neural underpinnings of these processes as well as the relation between them is lacking in the toddler and preschool years, due to significant challenges in acquiring quality neuroimaging data. Here, we use functional near‐infrared spectroscopy (fNIRS) to characterize neural activation in the frontal cortex for speech and gestures in toddlers at ages 2 and 3 years. We describe general patterns across both age groups, different patterns of neural activation in the frontal lobe in these two age groups, and preliminary evidence of a relation between neural activity for speech and gestures in 2‐year‐olds and language abilities at age 3.

fMRI studies support a shared neural basis for gesture and speech processing in adults. Specifically, overlapping activation for meaningful speech and gestures is seen in left inferior frontal gyrus (Andric et al., 2013; Redcay, Velnoskey, & Rowe, 2016; Straube, Green, Weis, & Kircher, 2012; Xu, Gannon, Emmorey, Smith, & Braun, 2009) as well as posterior temporal regions either within the left (Redcay et al., 2016) or right hemisphere (Andric et al., 2013) or across both hemispheres (Straube et al., 2012; Xu et al., 2009). These findings support the hypothesis that a shared neural basis for speech and gesture may underlie their shared cognitive bases and also their developmental co‐emergence (Bates & Dick, 2002). What is less clear is when and how this shared neural representation emerges in development.

The toddler years are an important time to study the relation between speech and gesture including their neural underpinnings. Between 9 and 12 months of age, infants begin production of communicative gestures and spoken words, with production of gestures generally preceding production of words (Woodward & Jesus Guajardo, 2002). More advanced elements of gesture such as recognitory (i.e., actions associated with objects) and communicative gesture continue to develop in conjunction with speech, until more complex speech and grammar arise at around 24–30 months (Bates & Dick, 2002). In the toddler years, gesture production is predictive of both qualitative and quantitative aspects of language in 3‐year‐olds (Goldin‐Meadow, 2015), and gestures, (including dietic gestures, such as pointing to a referent object while saying a word, as well as iconic gestures to illustrate motor acts, like hitting a baseball, or to indicate object attributes, like a building being tall) continue to augment speech comprehension in children and adults. The largest effects for improvement of speech comprehension via accompanying gestures are seen in studies of school‐aged children (Hostetter, 2011). These findings indicate a potential role for scaffolding of language development via gestures, particularly while verbal communication is emerging in the toddler years.

Compared to behavioral indicators of communicative development, less is known about the neural development of language or gestures in the second and third year of life. Cross‐sectional studies in the first 2–3 years of life highlight changes in neural activity for speech sound processing. Specifically, there is an increasing leftward lateralization for native speech sounds in the second year (Fava, Hull, & Bortfeld, 2014). Similarly, toddlers demonstrate more distributed patterns of activation (through EEG and ERP studies), including prefrontal cortex, that become more focal with age and experience (Coffey‐Corina & Neville, 1997; Mills, Coffey‐Corina, & Neville, 1997; Mills, Coffey‐Corina, & Neville, 1993; Mills et al., 2004; Redcay, Haist, & Courchesne, 2008). This frontal activity may serve an important role during language acquisition but not language use, consistent with the skill‐learning hypothesis (i.e., interactive specialization, Johnson, 2011).

Even less work has examined the neural bases of gestural development. Already by 8 months infants discriminate between congruent and incongruent point gestures via the N400 over right temporal cortex (Gredeback & Melinder, 2010), suggesting emergence of a neural basis for gesture processing by this time. More work has examined the neural correlates of social stimuli, which are an important component of both gesture and language processing (e.g., Kuhl, 2010; Ramirez‐Esparza, Garcia‐Sierra, & Kuhl, 2017). Infants as young as 4–5 months engage similar medial and left lateral prefrontal responses to gestures signaling communicative intent, such as eye contact (Grossmann, Parise, & Friederici, 2010) and joint attention (Grossmann & Johnson, 2010). Already by 5 months of age infants demonstrate specialized responses to biological motion (i.e., hand and eye movements) over posterior temporal (Lloyd‐Fox et al., 2009) and lateral inferior frontal (Lloyd‐Fox, Blasi, Everdell, Elwell, & Johnson, 2011) regions, similar to adults. These data suggest specialization for social and communicative stimuli early in the first year of life. Interestingly, research on auditory social stimuli reveals a more complex pattern of developmental specialization in infants, with selectivity for nonsocial (environmental sounds) stimuli compared to social (human vocal sounds) in the first year with selectivity to social stimuli emerging robustly by the second year of life (Lloyd‐Fox et al., 2017).

Only a limited number of studies have directly investigated whether there are shared regions for processing gestures and speech. In one, unlike adults, infants demonstrate nonoverlapping activation within left lateral prefrontal cortex to eye gaze and speech (one's own name, Grossmann et al., 2010). These data suggest visual and auditory modalities may be more distinct in infants, particularly within prefrontal cortex. However, a study investigating the neural response to gestures and speech at both 18 and 26 months of age demonstrated greater similarities in speech and gesture processing at 18 months than at 26 months (Sheehan, Namy, & Mills, 2007). Specifically, while 18‐month‐olds showed an N400 component to a picture mismatched to either gestures or speech, 26‐month‐olds only showed the N400 component when the picture was preceded by mismatched speech (as opposed to mismatched gesture). These data suggest a developmental change in the neural bases of gesture processing such that gestures may be part of the same communicative system as spoken language in the toddler years but may show greater distinctions at other points in development (Bates & Dick, 2002). Whether overlapping or distinct prefrontal regions would be engaged for words and gestures at a time when they begin to serve a common communicative function, however, remains an open question due to the lack of studies addressing the neural correlates of speech and gestures in toddlers. fNIRS methods can provide greater spatial resolution to disentangle questions of neural overlap in this age group.

The present study uses fNIRS to measure localization and lateralization of neural activity in frontal cortex during communicative and noncommunicative gesture and speech perception in 2‐ and 3‐year‐olds. In Study 1, we compare data from typically developing 2‐ and 3‐year‐olds and hypothesize that (1) both groups will show left lateral prefrontal activation to both communicative speech and gestures, as is consistent with adult work (Andric et al., 2013; Straube et al., 2012; Xu et al., 2009), (2) that lateral and medial prefrontal activation will be greater in 2‐ than 3‐year‐old children (consistent with extant ERP and fMRI studies and the skill‐learning hypothesis; (Johnson, 2011)), and (3) localization of speech and gesture processing at both ages will show both overlapping and spatially distinct regions of activation, with greater distinction at age 3. In Study 2, we analyze fNIRS data collected at age 2 in relation to behavioral data collected in the same children at age 3, including data from a sample of toddlers with both typical language development and language delays. We hypothesize that greater spatial distinction between regions of activation for speech and gestures represents a more mature developmental pattern, and thus will be associated with greater verbal abilities at age 3.

## 2 METHOD

This study was approved by an NIH Institutional Review Board. Consent was provided by parents or guardians for all participants. All participants were enrolled in a larger longitudinal study focused on developmental outcomes in toddlers with early language delays and those with typical development. This study included visits at 18 months (± 3 months), 24 months (± 3 months), and 36 months (± 3 months). At those visits, diagnostic and language/communication evaluations were completed, and fNIRS measurements were sometimes completed, dependent on time and toddler cooperation. The specific inclusion and exclusion criteria for participants in Studies 1 and 2 are further detailed below.

### 2.1 Study 1

Study 1 used cross‐sectional comparison of typically developing toddlers to examine variability in neural responses to language and communication in typical development. Participants were 27 typically developing toddlers, including 2‐year‐olds (mean age = 24.36 months, *n* = 15, 6 females) and 3‐year‐olds (mean age = 36.12 months, *n* = 12, 5 females) who had completed the fNIRS task at either the 24‐month or 36‐month visit as part of their enrollment in a longitudinal study of language development (see Table 1). An additional six children attempted the fNIRS task, with one being excluded due to equipment failure (age = 36 months) and five children excluded because they were not able to tolerate the headband (three children at 24 months, two children at 36 months). While one child had completed the fNIRS task at both the 24‐ and 36‐month visits, only data from the 36‐month visit were used for Study 1 for this child; all other toddlers had only completed the task at one of the visits. The larger longitudinal study in which these toddlers were involved measured language abilities in both typically developing toddlers and toddlers with early language delays; all toddlers included in Study 1 were from the typically developing group, as the focus of Study 1 was on typical patterns of neural activity for linguistic and communicative stimuli. Inclusion for the TD group for both the larger longitudinal study and for Study 1 required (a) no impairment or delays requiring intervention, (b) no first‐degree relative with a diagnosis of autism spectrum disorder, and (c) nonverbal and verbal scores within 1.5 *SD* of the mean on the Mullen Scales of Early Learning (MSEL, Mullen, 1995). All Study 1 participants were exposed to English as the primary language spoken at home, were born at or later than 36 weeks gestation, and were not reported to have genetic disorders or motor delays.

**Table 1:** Demographics for Study 1 (cross‐sectional sample)

	*n*	Age in months (*SD*) [range]	MSEL ELC (*SD*) [range]	Male: Female
Two‐year‐olds	15	24.36 (0.72) [23.04–26.52]	114.4 (11.3) [95–134]	10:5
Three‐year‐olds	12	36.12 (0.6) [34.8–37.44]	119.9 (13.1) [94–137]	7:5

Abbreviation

ELC, early learning composite.

### 2.2 Study 2

In Study 2, we report fNIRS results from all toddlers enrolled in the longitudinal study who had usable fNIRS data acquired at age 2 and language outcome data at age 3. This study was focused on relations between continuous language outcomes and fNIRS measurements across a variety of outcomes, and thus was not limited to participants from the typically developing group. Therefore, Study 2 included typically developing toddlers (*n* = 16) and language delayed toddlers (*n* = 2), for a total sample of 18 toddlers (seven females, mean age = 24.48 months). This sample includes one child whose data from the 3‐year visit was used in Study 1; fNIRS data from this child's 2‐year visit were used in Study 2 (see Table 2). The two toddlers with language delay included in Study 2 had initial evaluation at 18 months and had (a) both receptive and expressive language scores in the Very Low range (T‐scores ≤ 30) on the MSEL, (b) limited use of spoken words, and (c) no known medical issue responsible for delays. For all participants in Study 2 (including those with language delay), English was the primary language spoken at home as ascertained by parent report. Exclusion criteria for all toddlers in the larger longitudinal study as well as in Study 2 were prematurity (i.e., born before 36 weeks), known genetic disorder, or significant motor delay.

**Table 2:** Demographics for Study 2 (longitudinal sample)

Two‐year visit				
Age in years	Visual reception	Fine motor	Receptive language	Expressive language
24.48 (0.72) [23.04–26.52]	59.1 (7.5) [43–80]	51.4 (8.7) [39–68]	58.4 (6.9) [47–69]	55.1 (12.5) [38–73]

Three‐year visit				
36.6 (0.72) [34.8–38.4]	65.1 (9.3) [43–80]	50.4 (10.0) [32–71]	58.5 (9.8) [37–75]	58.5 (6.5) [43–68]

Note

Scores represent T‐scores on subtests of the Mullen Scales of Early Learning, which have a mean of 50 and a standard deviation of 10.

### 2.3 Stimuli

**Figure 1:**
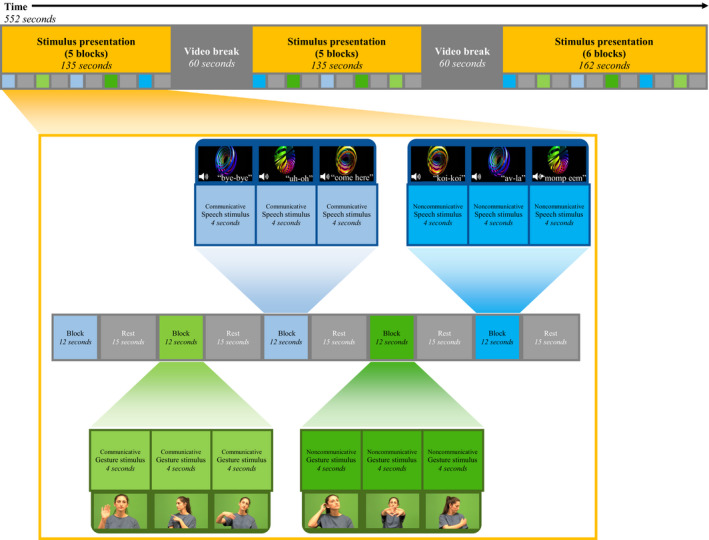
Stimulus presentation timeline. This figure illustrates the general timeline of the paradigm viewed by participants during the data collection session and examples of the four stimulus types used in the study. Examples of gesture and speech stimuli are included. The order of blocks was randomized by participant

Stimuli were identical across studies 1 and 2. Two types of gesture stimuli and two types of speech stimuli were presented on a 13” laptop screen using EPrime, which also sent triggers at the beginning of each block to the fNIRS computer. Gestures included both communicative gestures (e.g., wave, point) and noncommunicative gestures (e.g., tucking the hair behind the ear). Gesture stimuli were presented as silent videos of a woman facing a camera and making each of the gestures, and were piloted and edited within our laboratory. Speech stimuli included both words (e.g., “bye‐bye”) and nonwords (e.g., “ee‐vay”). Speech stimuli were presented while a screensaver shape moved across the laptop screen to maintain visual engagement. Each type of stimulus was presented in block format, with each block including three, 4 s, consecutive exemplars of each stimulus type for a total of 12 s of continuous stimuli per block, followed by 15 s of rest. Each of the four stimulus types were presented in four blocks, for a total of 16 blocks, with conditions randomly distributed across the presentation. Two 1‐min videos from a popular children's show were presented after five to six blocks of stimuli to engage children and reduce boredom and movement. A timeline of the task and examples of condition stimuli are presented in Figure 1.

Children were videotaped using the video camera internal to the presentation laptop, and videos were coded for time looking at the screen, fussing, and significant movements. Data from the gesture conditions were used only if the child was looking at the screen for at least 50% of the 12‐s block, while data from the speech conditions were used as long as children were calm and still.

### 2.4 Procedure

Each participant completed the MSEL (Mullen, 1995), a standardized developmental measure with standardized scores for gross motor ability, two areas of nonverbal cognitive development (visual reception and fine motor) and two areas of language development (receptive language and expressive language). The MSEL was completed at all study visits in the larger longitudinal study, including both the 24‐month and 36‐month visits described here. After completion of the MSEL as well as other behavioral measures that were not a focus of this particular study, participants were seated at a child‐size chair in front of a laptop screen and were shown an entertaining video while the fNIRS headband was placed. The headband was secured with athletic wrap for each child and signal quality was optimized via the real‐time fNIRSOFT Cobi package (Ayaz, 2005). Specifically, optode light intensity was calibrated to reduce both signal saturation and weak signals across the entire headband. This involved an auto‐calibration process within the Cobi package whereby gain is reduced if intensity surpasses the acceptable range for the photodetectors (Ayaz et al., 2011) as well as reapplication or adjustment of the headband if signal intensity indicated reduced skin contact. Stimuli were presented via EPrime 2.0, which was also used to send pulses to the fNIRS computer indicating presentation of stimuli. Once the experiment began, the experimenter interacted with the child only to orient them to the screen if they were looking away. One‐minute entertaining children's videos were interspersed to reorient children and decrease fatigue.

### 2.5 FNIRS imaging and processing

Data collection and processing were identical across studies 1 and 2. Data were collected with a continuous wave fNIRS system (FNIRS Devices LLC, MD) at wavelengths of 730 and 850 nm across 4 sources, 10 detectors, yielding 16 data channels, with a constant source‐detector separation of 2.5 centimeters and a sampling rate of 2 Hz. This system has all sources and detectors integrated within a single silicon band, and because it is nonfiber‐based can be worn comfortably on the forehead (see Figure S1.) The band was centered at FPZ, based on the international 10–20 transcranial positioning system. Data were processed using fNIRSOFT (Ayaz, 2010). We applied the SMAR (Sliding‐Window Motion Artifact Rejection) filter to the raw light density data, which in addition to removing motion artifact removes data where the signal is either saturated or faint (Ayaz, Izzetoglu, Shewokis, & Onaral, 2010), then median filtered (order 20). Then, we applied a 0.2 Hz finite impulse response (FIR) linear phase low‐pass filter, order 20. The function of the FIR low‐pass filter is to remove physiological noise while maintaining the signal expected for a block design (e.g., Bogler, Mehnert, Steinbrink, & Haynes, 2014; Naseer & Hong, 2013; Shin, Müller, & Hwang, 2016; Taga, Watanabe, & Homae, 2018). Within FNIRSOFT, The Modified Beer–Lambert law was used to calculate changes in oxy‐ and deoxyhemoglobin from baseline, with differential path length set at 6 for all participants. Oxy‐ and deoxyhemoglobin data were further processed to remove motion artifact using a Correlation Based Signal Improvement (CBSI) filter with standard settings, which corrects for parallel changes in oxy‐ and deoxyhemoglobin signals (Cui, Bray, & Reiss, 2010). Finally, signal drift was corrected via first order linear detrending applied at each channel. Motion artifacts were therefore addressed by removing data with very high intensities (i.e., SMAR filter, standard settings), and by correcting sudden signal changes via median filter, low‐pass filter, and CBSI filter.

For participants who tolerated the headband, data loss was driven by either not looking at the screen or loss during fNIRS processing via the SMAR filter (see Table S1). All participants who tolerated the headband contributed data to this dataset, and linear mixed effects modeling as described below was used to account for differences in trials completed by participant. Across studies 1 and 2, from the total potential 7,680 data points (30 participants, 16 blocks, 16 optodes), 58% of data remained (4,486 data points). After data that were contaminated with artifact were removed, differences in data loss by age, mode, and condition were investigated. Age did not predict data loss (*t* = −0.47, *p* = .64). Gestures and nonmeaningful stimuli were associated with less artifact removal when compared to speech and meaningful stimuli (*t* = −2.54, *p* = .011; *t* = −2.61*, p* = .009). However, Levene's test for heteroscedasticity showed that the assumptions of equal variances were met for the effects of mode (gestures vs. speech) and condition (meaningful vs. nonmeaningful) at all 16 channels (all *p's*>0.05).

Z‐scores were then calculated for each measurement of oxy‐ and deoxyhemoglobin density relative to the mean and standard deviation of oxy‐ and deoxyhemoglobin values for each individual toddler at each channel across all trials with useable data. Thus, a given z‐score value indicates the number of standard deviations from the mean for that measurement (i.e., from the 24 samples of data from each 12 s trial sampled at 2 Hz) in relation to all other measurements occurring within the same channel for the same individual across all conditions, including rest, after initial data processing. Converting measurements to z‐scores reduces the impact of individual differences in differential path length factor, which can vary with age, skull characteristics, and skin tone (Moriguchi & Hiraki, 2013). Z‐scores were calculated for all data for a given individual after all data filters were applied. After calculation of z‐scores, mean z‐scores for each block were calculated only for those blocks including at least 20 consecutive samples (10 s). These values were then averaged by condition type at each channel, providing each participant a mean oxy‐ and deoxyhemoglobin value for each condition at each channel. The use of a block average as an outcome measure, rather than coefficients reflecting a modeled hemodynamic response (e.g., Issard & Gervain, 2018) as an outcome measure for block designs is an approach that has been taken in other fNIRS studies with children (Monden et al., 2015; Soltanlou et al., 2017; Sulpizio et al., 2018). Unlike modeling a hemodynamic response, block averaging does not depend on the assumption that hemodynamic responses of interest must follow a particular function. This is especially important in infants and young children, as assumptions regarding the shape of the hemodynamic function are generally based on adult fMRI literature and may not be valid or may be driven differently by study design (Issard & Gervain, 2018).

Analyses in Study 1 included mixed effects modeling in R (Bates, Machler, Bolker, & Walker, 2015) with subject ID as a random effect (allowing HbO changes at the trial‐level to be the dependent variable while accounting for different patterns across participants). Condition (i.e., words vs. nonwords, communicative vs. noncommunicative gestures) was modeled as a fixed effect, with both predicting changes in mean HbO z‐scores at each of the 16 channels. Age, coded as a categorical variable, was entered as an interaction term with condition and mode in predicting HbO changes in order to determine the differences between 2‐ and 3‐year‐olds, with both the effects of age and condition alone entered first. When there was no significant interaction between age and condition, the effect of condition alone was evaluated still controlling for the effect of age. For longitudinal analyses in Study 2, general linear models were used to predict MSEL Receptive Language and Expressive Language T‐scores, standardized scores with a mean of 50 and a *SD* of 10, at age 3 years, with z‐score change at each channel as the predictor while controlling for language T‐scores at age 2 (e.g., MSEL Receptive Language or Expressive Language T‐score at age 2). We controlled for language T‐scores at age 2 by including them as a covariate predicting language T‐scores at age 3, thus investigating statistical significance of z‐score changes at each channel after accounting for variance related to language at age 2. Because all statistics were conducted at each of 16 channels, the Benjamini‐Hochberg procedure was used to test for significance within the context of multiple comparisons.

## 3 RESULTS

### 3.1 Study 1

#### 3.1.1 Main effects of communicative mode: speech versus gesture

**Figure 2:**
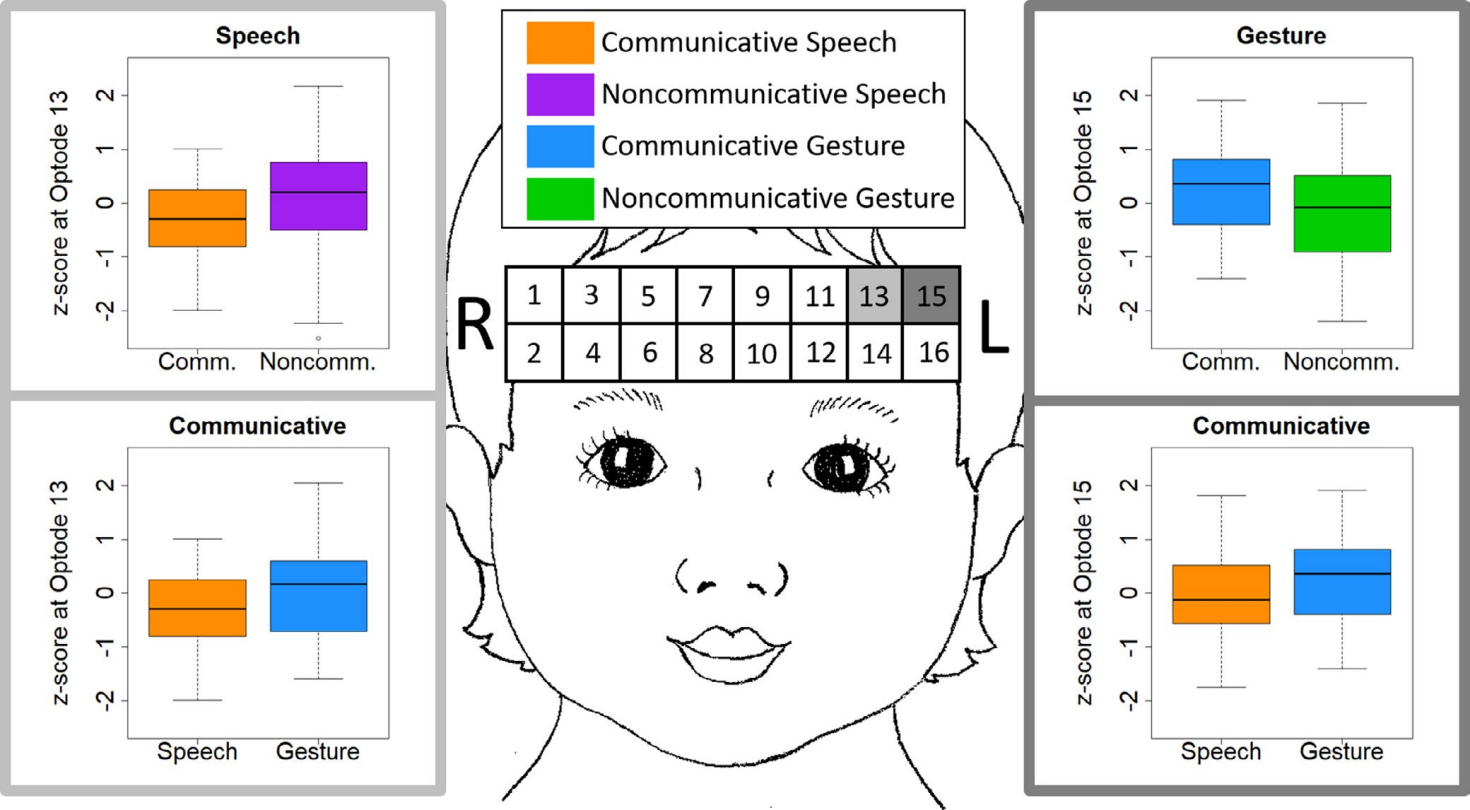
Neural discrimination of communicative and noncommunicative speech and gesture in left lateral frontal cortex. All interactions plotted were significant predictors of oxyhemoglobin levels at the designated optode. Error bars show Standard Error of the Mean

Hemoglobin values varied between communicative speech and gesture in the left lateral channels (see Figure 2, Figure S2). Specifically, oxyhemoglobin values were higher for gestures than speech in channels 13 (for combined sample, *t* = 2.21, *p* = .030; for 2‐year‐olds, *t* = 2.15, *p* = .036; for 3‐year‐olds *t* = 1.01, *p* = .32) and 15 (for combined sample, *t* = 2.59, *p* = .011; for 2‐year‐olds *t* = 1.49, *p* = .14; for 3‐year‐olds *t* = 2.18, *p* = .034). There was no interaction between age and communicative mode at either channel (for 13, *t* = 0.72, *p* = .47; for 15, *t*=−0.59, *p* = .55). These main effects continued to be significant after accounting for multiple comparisons.

#### 3.1.2 Interaction with age: speech versus gesture

Two‐ and 3‐year‐olds showed different neural discrimination of communicative speech and gestures in the right lateral region (*t* = −2.1, *p = *.040). Specifically, 2‐year‐olds showed higher levels of oxyhemoglobin for speech compared to gestures in channel 4 (*t* = −2.4, *p* = .023), while 3‐year‐olds did not show differentiation (*t* = 0.62, *p* = .54). This interaction with age was not significant after accounting for multiple comparisons.

#### 3.1.3 Main effects of condition: communicative versus noncommunicative

Oxyhemoglobin values varied between the communicative and noncommunicative conditions (both speech and gesture) in the left lateral regions. Specifically, noncommunicative speech (i.e., nonwords) was associated with greater levels of oxyhemoglobin than communicative speech at channel 13 (for combined sample, *t* = 3.0, *p* = .003; 2‐year‐olds *t* = 1.02, *p* = .31; 3‐year‐olds *t* = 3.35, *p* = .001). The interaction between condition and age was not significant at this channel (*t* = 1.94, *p* = .053). On the other hand, communicative gestures were associated with higher levels of oxyhemoglobin than noncommunicative gestures in channel 15 (for combined sample, *t* = −2.7, *p* = .0080; for 2‐year‐olds *t* = −0.6, *p* = .53; for 3‐year‐olds, *t* = −3.23, *p* = .0022, see Figure 2, Figure S2.) The interaction between age and condition was not significant for gestures (*t* = 1.62, *p* = .11). The main effect of condition continued to be significant after accounting for multiple comparisons.

#### 3.1.4 Interaction with age: communicative versus noncommunicative

**Figure 3:**
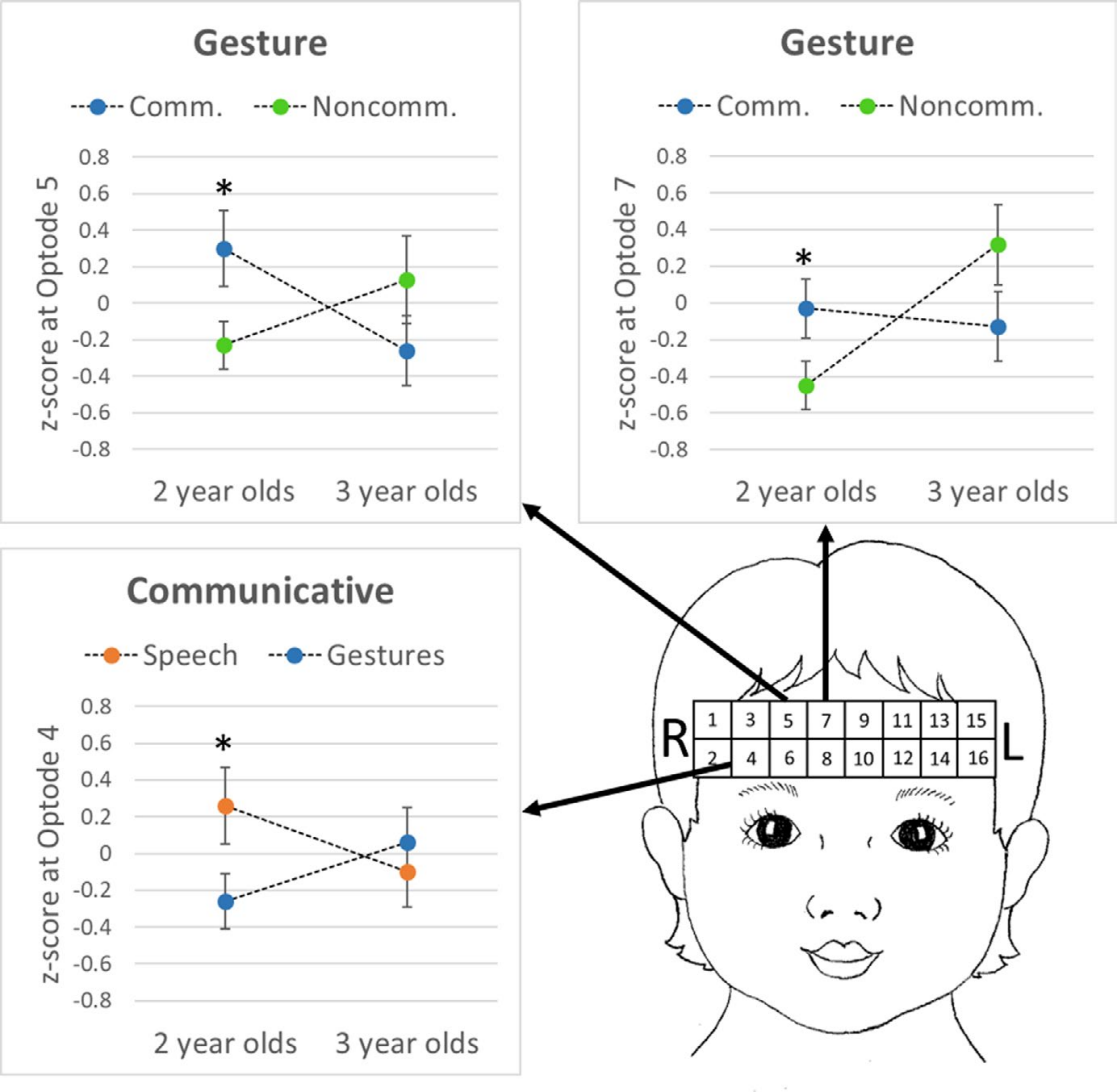
Differences in neural discrimination between age groups in right frontal cortex. All interactions shown are significant. *Indicates a significant effect (*p*<.05) of condition within that age group. Error bars show Standard Error of the Mean

There were no channels at which the interaction between age and condition (i.e., communicative and noncommunicative) was significant for speech stimuli. For gestures, age‐related differences were visible in the right medial frontal regions including channels 5 (*t* = −2.40, *p* = .019) and 7 (*t* = −2.45, *p* = .017, Figure 3). Specifically, 2‐year‐olds showed higher oxyhemoglobin levels for communicative gestures versus noncommunicative gestures (channel 5 *t* = −2.30, *p* = .027; channel 7 *t* = −2.09, *p* = .043) while 3‐year‐olds did not show neural differentiation of the two conditions (channel 5 *t* = 1.23, *p* = .23, channel 7 *t* = 1.54, *p* = .13). This differentiation specific to 2‐year‐olds is in contrast to neural differentiation of communicative versus noncommunicative gestures across the entire sample (i.e., both 2‐ and 3‐year‐olds), which was located in the left lateral cortex. The interaction with age was not significant after accounting for multiple comparisons.

### 3.2 Study 2

**Figure 4:**
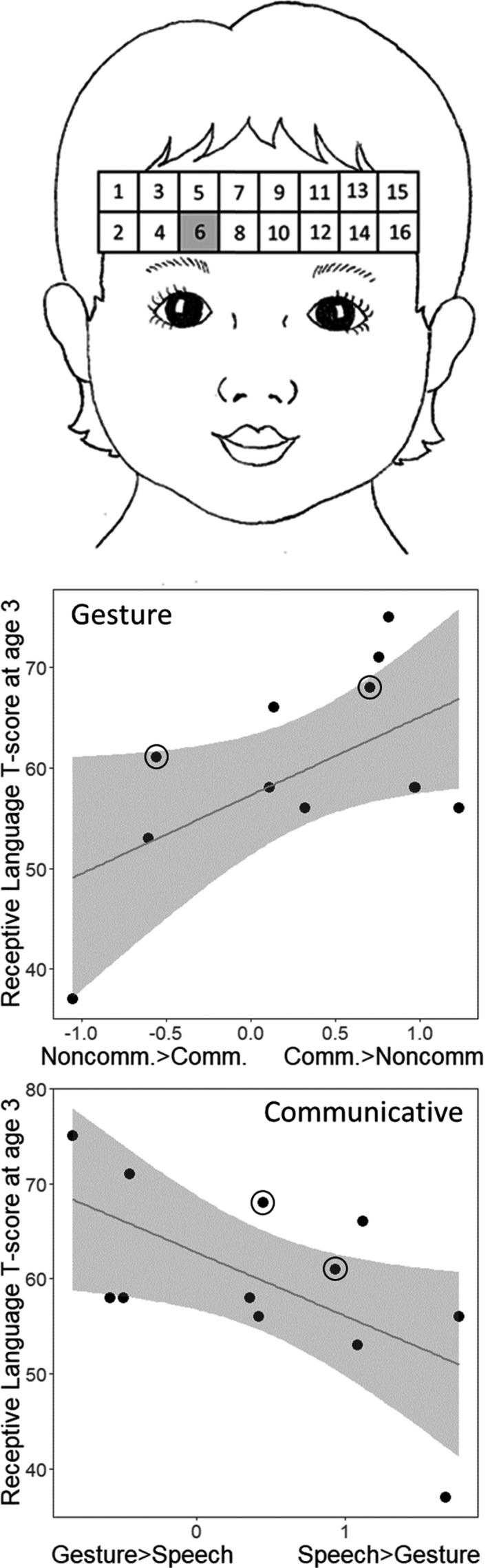
Neural differentiation patterns associated with later receptive language abilities. Circled data points indicate data from participants with early language delays. Greyed area represents 95% confidence interval

Changes in oxyhemoglobin related to both condition and mode in right medial frontal cortex predicted language scores at age 3 above and beyond verbal abilities at age 2 (see Figure 4). Specifically, the effect of mode (speech vs. gesture) at age 2 in right medial cortex predicted receptive language scores at age 3 above and beyond receptive language ability at age 2 (at channel 6, β = 0.06, *SE* = 0.03, *t* = 2.2, *p* = .034). Specifically, higher oxyhemoglobin levels for gestures versus speech in channel 6 predicted higher receptive language T‐scores at age 3. Second, the effect of condition (communicative vs. noncommunicative) for gestures alone at age 2 in right medial cortex predicted receptive language T‐scores at age 3 (at channel 6, β=−0.059, *SE* = 0.024, *t*=−2.5, *p* = .018). Specifically, greater oxyhemoglobin values for communicative gestures versus noncommunicative gestures in channel 6 were associated with higher receptive language at age 3. These effects were not significant after accounting for multiple comparisons.

## 4 DISCUSSION

The present study measured cortical activation patterns in the frontal lobe in toddlers during presentation of communicative and noncommunicative speech and gestures. Study 1 found cortical activation differences in response to communicative versus noncommunicative speech and gestures in toddlers, including activation differences that varied with age across 2‐ and 3‐year‐olds, in left lateral regions as well as right medial regions. Study 2 found that in 2‐year‐olds, including those with typical development and those with early language delays, patterns of activation specific to meaningful gestures within the right medial frontal lobe were associated with higher receptive language abilities at age 3. These findings expand the literature on neural underpinnings of those developmental changes occurring in the previously understudied toddler years, and provide support for the potential use of functional brain activation during presentation of communicative stimuli as a marker for language outcomes.

In Study 1, 2‐ and 3‐year‐olds showed neural differentiation of communicative versus noncommunicative speech and gesture stimuli as well as differentiation of communicative speech versus gestures in left lateral frontal regions. Patterns of neural differentiation at this age are consistent with general findings that show neural differentiation of communicative and noncommunicative cues in infancy (Bakker, Kaduk, Elsner, Juvrud, & Gustaf, 2015; Dehaene‐Lambertz, Dehaene, & Hertz‐Pannier, 2002; Friedrich & Friederici, 2005; Gredeback & Melinder, 2010; D. L. Mills et al., 2004). Location of findings within the left lateral frontal regions as opposed to the right hemisphere is also consistent with literature showing greater left hemisphere activation for communicative stimuli in the first year of life (Conboy & Mills, 2006; Dehaene‐Lambertz et al., 2002) and in adults for both speech and gesture (Bates & Dick, 2002; Redcay et al., 2016). Greater activation in left lateral frontal regions in response to meaningful gesture versus meaningful speech highlights the importance of gestures in communicative development in this age range. Furthermore, these data demonstrate continuity in the neural bases of speech and gesture over development.

Age‐related differences in activation were located in the right medial frontal regions, with 2‐year‐olds showing neural differentiation of gestures versus speech and communicative versus noncommunicative gestures and 3‐year‐olds showing no such differentiation. This is in line with research showing activation of right frontal regions in young infants for communicative versus noncommunicative facial gestures (Grossmann, Lloyd‐Fox, & Johnson, 2013), and extends this pattern to an older age in response to manual gestures. Medial prefrontal differentiation in 2‐year‐olds but not 3‐year‐olds in this study might be explained in the context of interactive specialization or skill‐learning hypotheses, in which case frontal regions play a greater role during acquisition of a skill (i.e., communicative gestures) than during later use. Alternatively, age‐related differences in neural differentiation of gestures within the right medial frontal areas, as seen here, may be due to differences in attentional demands for gesture processing at those ages. In adults and children, activation of right medial frontal regions has been associated with attentional mechanisms important for speech processing (Arredondo, Hu, Satterfield, & Kovelman, 2017; Konrad et al., 2005; Kristensen et al., 2018). Activation in right medial frontal cortex may be especially important for regulation of attention to social stimuli, and integration of ventral and dorsal attention networks has been localized to this region in adults (Corbetta, Patel, & Shulman, 2008). Therefore, neural differentiation of gestures in right medial cortex in 2‐year‐olds but not 3‐year‐olds may reflect differential activation of attentional networks for the gestures used in this study, which could represent the neural underpinnings of the prioritization of communicative gestures at this age. Notably, however, this finding of increased prefrontal activity in toddlers did not extend to speech stimuli. The lack of neural differentiation of communicative and noncommunicative speech is in contrast to activations in right frontal regions during speech processing in awake infants (Dehaene‐Lambertz et al., 2002) and sleeping toddlers (Redcay et al., 2008).

Both 2‐ and 3‐year‐olds demonstrated differential processing of communicative content within speech and gesture domains. Interestingly, the discrimination of communicative versus noncommunicative stimuli engaged adjacent but distinct channels over left lateral frontal cortex. Contrary to our hypothesis that shared processing would be greater in 2‐ compared to 3‐year‐olds, these findings did not show any interactions with age. Rather, these data are consistent with findings from younger infants that auditory and visual communicative stimuli differentially engaged nearby but distinct regions of left lateral prefrontal cortex (Grossman et al., 2010). Given that there is considerable data supporting overlapping regions in left posterior temporal regions as well as left inferior frontal regions for shared activation for both gestures and speech in older children and adults (Dick, Goldin‐Meadow, Solodkin, & Small, 2012; Redcay et al., 2016; Straube et al., 2012; Xu et al., 2009), these data suggest a process of gradual increasing integration between gesture and speech with age (beyond our sample).

In line with this conceptualization, Study 2 showed that differential activation in the right medial frontal regions for communicative and noncommunicative gestures as well as communicative gestures and communicative speech at age 2 was associated with higher receptive language abilities at age 3. Importantly, these patterns varied in their direction in relation to age‐related differences. Specifically, while 2‐year‐olds as a group showed higher oxyhemoglobin levels for speech versus gestures in this region in Study 1, it was the opposite pattern (i.e., higher levels for gestures vs. speech) that positively predicted higher receptive language at age 3. However, 2‐year‐olds as a group also showed higher activation for communicative versus noncommunicative gestures, and it was the strength of this pattern that was positively associated with receptive language abilities at age 3. This latter finding, in particular, is in line with the interactive specialization hypothesis, which proposes that recruitment of brain regions outside those regions associated with specialized processing in the adult brain promotes emergence of cognitive abilities, including language (Johnson, 2011). Furthermore, that greater oxyhemoglobin for communicative gestures (both when compared to noncommunicative gestures and communicative speech) was related to later language abilities also provides evidence of developmental scaffolding of language development by gesture at the neural level. The fact that right medial rather than left lateral regions predicted language outcomes emphasizes the role of top down mechanisms related to awareness and attention in learning social communicative stimuli generally, and is in itself in line with the interactive specialization hypothesis.

### 4.1 Limitations and future directions

A primary limitation of this study is the small sample size, necessitating replication with larger samples for full integration with the literature. When correcting for measurement and analysis across multiple channels within this sample, the main effects from Study 1 are preserved, while age and predictive effects should be interpreted as exploratory. Given the potential for nonrandom noise to drive effects in fNIRS data and the variety of processing choices currently used to address these concerns (Huppert, 2016), it will also be important to investigate these patterns using other processing options, including those dependent on use of short spatial separation channels, which allow for reduction of physiological noise outside of use of bandpass filters. Second, the configuration of the fNIRS sensors within a frontal headband limited investigation of functional activation to frontal and prefrontal regions of the brain. While this allows for quantification of activation within brain regions important for speech processing and social attention, it notably excludes measurement over the temporal speech regions as well as temporo‐parietal regions involved in gesture processing. In addition, the placement of optodes across subjects is kept constant in spite of different head shapes and sizes. Therefore, the specific underlying structures measured here are inferred based on placement, but future research paired with structural MRI data can clarify how individual differences in underlying anatomy may contribute. The band was chosen for the present study due to its ease of application, which was particularly important because of compliance issues involved in neuroimaging with toddlers. Future fNIRS language studies should ideally incorporate a wider array over the scalp to ensure that key language areas are examined. Third, the distribution of receptive and expressive language abilities at 3 years was skewed toward average and above average abilities, making interpretation of possible predictions from functional activation at 2 years challenging in children with below average language abilities or those with language impairments. While the present sample was supplemented with data from toddlers with early indicators for language delay, a wider range of developmental abilities would afford a fuller picture of how early neural measures relate to a variety of language outcomes. In spite of these limitations, this study is a significant step forward for the literature because of the age of the sample, use of a functional task, and its utility as a preliminary investigation of how brain activity may predict language outcomes. The current study provides proof of concept that fNIRS can capture subtle changes in functional activity in the developing brain of toddlers, and that these changes may relate to ongoing emergence of language skills during this developmental stage.

## Conflict of Interest

None of the authors have any conflict of interest to report related to the completion of this work.

## Data Availability

The data that support the findings of this study are available from Elizabeth Smith at esmith23@umd.edu upon reasonable request.
