# Supplementary figures and images for: Functional near‐infrared spectroscopy in toddlers: Neural differentiation of communicative cues and relation to future language abilities
Source: Dev Sci. 2020 Mar 20;23(6):e12948. doi: 10.1111/desc.12948 (PMC7685129; doi:10.1111/desc.12948)

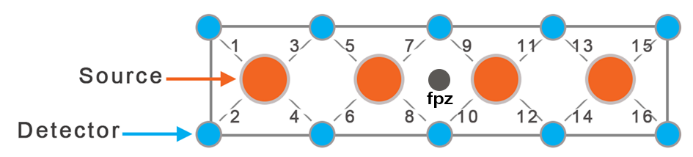

Supplement: Supplementary file 1 [file DESC-23-e12948-s001.tif]

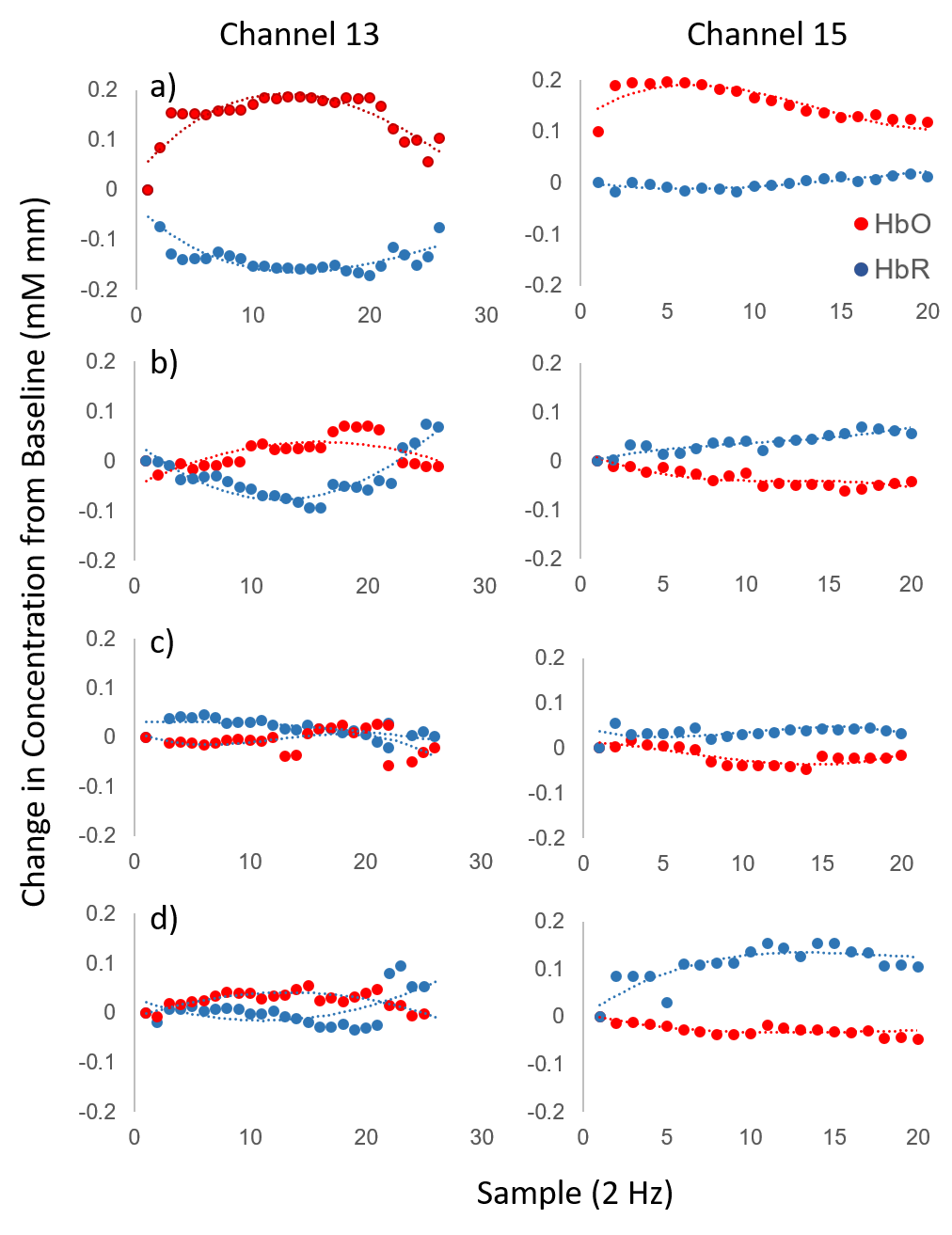

Supplement: Supplementary file 2 [file DESC-23-e12948-s002.tif]
