# Supplementary material for: Functional near‐infrared spectroscopy in toddlers: Neural differentiation of communicative cues and relation to future language abilities
Source: Dev Sci. 2020 Mar 20;23(6):e12948. doi: 10.1111/desc.12948 (PMC7685129; doi:10.1111/desc.12948)
Supplement: Supplementary file 3 [file DESC-23-e12948-s003.docx]

|  | | **Words** | **Nonwords** | **Gestures** | **Movements** | **Total** |
| --- | --- | --- | --- | --- | --- | --- |
| **2 year olds** | **Survive SMAR filter** | **47%** | **56%** | **53%** | **61%** | **54%** |
|  | **Survive looking time** | **47%** | **56%** | **26%** | **31%** | **40%** |

|  | | **Words** | **Nonwords** | **Gestures** | **Movements** | **Total** |
| --- | --- | --- | --- | --- | --- | --- |
| **3 year olds** | **Survive SMAR filter** | **53%** | **61%** | **67%** | **66%** | **61%** |
|  | **Survive looking time** | **53%** | **61%** | **37%** | **43%** | **48%** |

Supplemental Table 1. Data loss by age and stimulus type. Percent data indicates percent of oxyhemoglobin measurements across all samples (including baseline and rest) and at all locations.
